# Particulate air pollution and cardiovascular disease mortality in Jiangsu Province, China: a time-series analysis between 2015 and 2021

**DOI:** 10.3389/fpubh.2023.1218479

**Published:** 2023-12-20

**Authors:** Fangyu Zhu, Hao Yu, Xikang Fan, Zhen Ding, Qingqing Wang, Jinyi Zhou

**Affiliations:** ^1^Department of Non-communicable Chronic Disease and Prevention, Jiangsu Provincial Center for Disease Control and Prevention, Nanjing, China; ^2^Department of Environmental Health, Jiangsu Provincial Center for Disease Control and Prevention, Nanjing, China

**Keywords:** air pollution, particulate matter, mortality, cardiovascular disease, time-series study, China

## Abstract

**Introduction:**

Previous time-series studies have revealed a positive association between particulate matter (PM) and acute cardiovascular effects. However, the evidence mostly comes from developed countries and regions, while the majority of air-pollution-related deaths occur in developing countries. To assess the effect of short-term exposure to PM on daily cause-specific cardiovascular disease (CVD) mortality in Jiangsu Province, China, we investigated 1,417,773 CVD deaths from 2015 to 2021 in Jiangsu.

**Methods:**

The city-specific association was estimated using generalized additive models with quasi-Poisson regression, and then, random effects meta-analysis was performed to estimate the pooled provincial-average associations between acute exposure to PM_2.5_ and PM_10_ and cardiovascular disease mortality. To test the independence of PM from gaseous pollutants, we fitted two-pollutant models. Mortality data were also stratified by sex, age, and region to investigate the modification of associations. The exposure-response (E-R) curve from each city was combined using meta-analysis to drive the provincial-level E-R curve.

**Results:**

The results showed that each 10-μg/m^3^ increase in the PM_2.5_ concentration was associated with a 0.723% [95% confidence interval (CI): 0.512, 0.935] increase in daily total CVD mortality, a 0.669% (95% CI: 0.461, 0.878) increase in CHD mortality, a 0.758% (95% CI: 0.584, 0.931) increase in stroke mortality, a 0.512% (95% CI: 0.245, 0.780) increase in ICH mortality, and a 0.876% (95% CI: 0.637, 1.116) increase in CI mortality. The corresponding increases in daily mortality rates for the same increase in the PM_10_ concentration were 0.424% (95% CI: 0.293, 0.556), 0.415% (95% CI: 0.228, 0.602), 0.444% (95% CI: 0.330, 0.559), 0.276% (95% CI: 0.026, 0.526), and 0.510% (95% CI: 0.353, 0.667), respectively. The association between PM and total CVD mortality remained significant after adjusting for gaseous pollutants. Females, older adults and districts with lower average PM levels are more sensitive, especially for PM_10_. The E-R curve for PM on CVD mortality is steeper at lower concentrations and flattens out at higher concentrations. The estimates remained generally consistent in sensitivity analyses when excluding the data during the COVID-19 pandemic period.

**Discussion:**

Our time-series study provides evidence of positive associations between acute exposure to PM_2.5_ and PM_10_ and total and cause-specific cardiovascular disease mortality in developing countries.

## 1 Introduction

Ambient air pollution, which is a complex mixture of particulate matter (PM) and gaseous pollutants, is the fourth leading risk factor for death worldwide (1, 2). Available global studies estimate the number of deaths caused by air pollution at between 6.7 million, 8.8 million, and 10.2 million (3–5).

According to the WHO’s air quality guidelines, 92% of the population worldwide lives in places that still exceed the standard for annual mean PM, and low- and middle-income countries tend to have higher levels of pollution (6). Cardiovascular disease (CVD) is the leading cause of disabilities and fatalities globally, accounting for 61.9% of pollution-related deaths (7). Substantial evidence links air pollution, particularly PM_2.5_, to a range of cardiometabolic risk factors, including hypertension, insulin resistance, diabetes, cardiac arrhythmias, and obesity (8, 9). From the perspective of mechanisms, inhaling PM exacerbates existing cardiovascular disease and promotes its development primarily through inflammation, translocation into the blood, and effects on the autonomic nervous system (10, 11). It is essential to estimate the health effects of PM on CVD mortality to inform policies aimed at reducing exposure to pollution and improving public health outcomes.

Previous time-series studies have revealed a positive association between PM and acute cardiovascular effects (12–15). A systematic review and meta-analysis reported that increases in PM_2.5_ (particulate matter <2.5 μm diameter) and PM_10_ (particulate matter <10 μm diameter) concentrations were associated with hospital admission and mortality for stroke (13). A time-series study conducted in 75 U.S. cities from 2000 to 2006 demonstrated that increases in PM_2.5_ concentration were associated with increased risks of CVD, myocardial infarction, and stroke mortality (14). A nationwide investigation in China also added to the evidence of the short-term health impact of PM_2.5_ on mortality from various cardiopulmonary diseases (15).

However, the evidence mostly comes from developed countries and regions, while 89% of air pollution-related deaths occur in developing countries. The magnitude of the association between PM exposure and adverse health outcomes is different across high and low air pollution levels (16, 17). In addition, most of the previous research in China was conducted in a single megacity, and different modeling approaches and potential publication bias make those results less representative and comparable (18, 19). Moreover, the exposure effects of pollutants on cardiovascular events can be the result of interactions between gases and particles. The independent effects of PM need to be clarified by considering gaseous pollution impacts (20).

Therefore, we conducted a multicity collaborative time-series study in Jiangsu, China, to provide more representative and robust evidence on the association between PM and cardiovascular disease mortality.

## 2 Materials and methods

### 2.1 Study area

**Figure 1:**
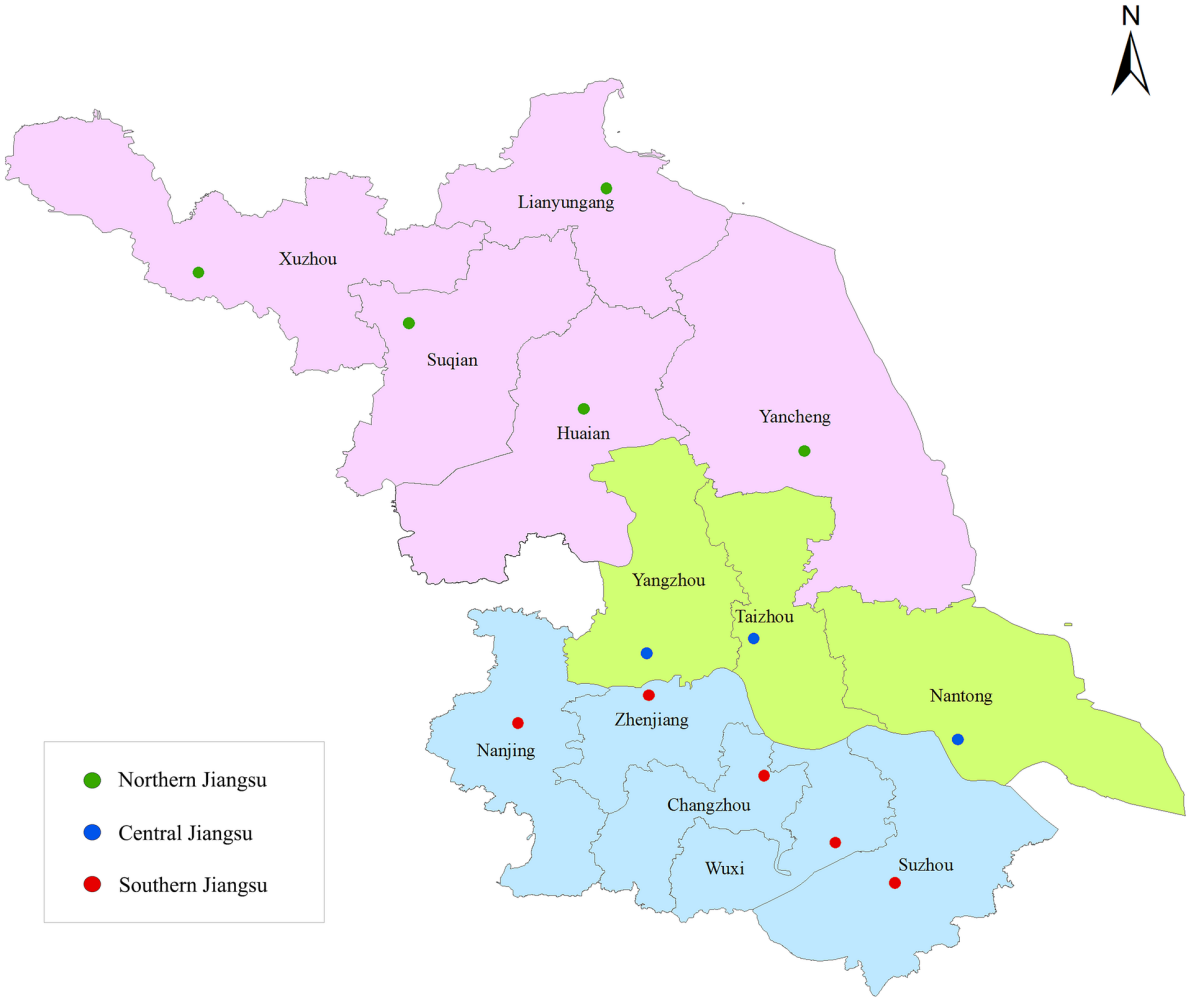
Locations of 13 cities in Jiangsu, China (green dots, Northern Jiangsu; blue dots, Central Jiangsu;ts, Southern Jiangsu).

Jiangsu Province is located on east coast of China, next to Shanghai. Jiangsu is one of the strongest economical regions in China, with a total area of 107,200 km^2^ and a permanent population of 84.75 million by the end of 2020. Jiangsu Province comprises 13 cities that are geographically categorized into three regions, namely Northern, Central, and Southern Jiangsu (Figure 1). The northern part is the warm-temperate humid and semi-humid monsoon climate, while the southern part is the subtropical humid monsoon climate. Economic disparities also exist among the three regions. Southern and Central Jiangsu had *per capita* GDPs of US$24,292 and US$17,433, respectively, while the *per capita* GDP in Northern Jiangsu stood at US$10,634 in 2018 (21).

### 2.2 Data collection

Daily mortality data for all 13 cities in Jiangsu Province from 2015 to 2021 were obtained from the Death Information Registration and Management System, which is operated by the Chinese Center for Disease Control and Prevention (China CDC). The underlying causes of death were determined by trained physicians and based on clinical records and related symptoms. The causes of death were classified and coded according to the International Classification of Diseases, 10th Revision (ICD-10) (22). The mortality data were subject to multilevel quality control procedures administered by the China CDC network at the county/district level, prefecture level, and provincial level and sampled and reviewed regularly to improve completeness and accuracy (23). We extracted daily cause-specific mortality data for cardiovascular diseases (CVD; codes I00-I99), coronary heart disease (CHD; codes I20-I25), stroke (codes I60-I69), intracerebral hemorrhage (ICH; codes I61), and cerebral infarction (CI; codes I63). A total of 1,417,773 deaths from cardiovascular diseases were included in our analysis.

Daily air pollution data from 206 environmental monitoring stations in all 13 cities were collected through the Air Pollution and Health Monitoring System administered by the China CDC. In our study, PM includes fine particulate matter PM_2.5_ and respirable particulate matter PM_10_, and gaseous pollutants include sulfur dioxide (SO_2_), nitrogen dioxide (NO_2_), carbon monoxide (CO), and ozone (O_3_).

For PM_2.5_, PM_10_, SO_2_, NO_2_, and CO, daily data were 24-h average concentrations, and for O_3_, they were 8-h averages. The system also collected meteorological data, including daily average temperature and daily average relative humidity (RH), for each city. Two cities (Nantong and Taizhou) did not carry out pollution monitoring in 2015, and thus, the data for these two cities in that year were excluded from our analysis.

### 2.3 Statistical analyses

We applied generalized additive models (GAMs) with quasi-Poisson regression to estimate the city-specific association between acute exposure to PM and cardiovascular disease mortality. Confounders included in the models are as follows: (1) a natural cubic smooth function with 7 degrees of freedom (df) per year for controlling long-term trends in daily mortality, (2) a day-of-week variable for possible variations in a week, (3) and a natural spline function with 6 df for temperature and 3 df for RH to control for confounding effects of weather conditions. We then applied random effects models to calculate the pooled estimates and their 95% confidence intervals (CI) of the provincial-average association as the percentage change in mortality per 10-μg/m^3^ increase in PM concentration. Single-day lagged concentrations and multiday moving averages for PM concentration within 4 days were examined. Lag days produced the largest effect estimates and were subsequently used in further analysis.

To test the independence of PM_2.5_ and PM_10_ from co-pollutants, we fitted two-pollutant models. First, we calculated Spearman correlation coefficients to investigate the associations between pollutants and meteorological factors. For gaseous pollutants that had a correlation with PM exceeding 0.5, we considered this as a moderate to strong correlation and then applied it in two-pollutant models. The association was considered to be independent if the pooled estimate remained significant after adjusting.

Mortality data were stratified by sex (male or female) and age (5–64 years, 65–74 years, and > =75 years) to investigate the modification of associations between PM and cause-specific CVD mortality. We also derived region-specific estimates by meta-analysis to investigate potential region-level effect modifiers. Statistically significant differences in associations between the strata were tested by using a paired z-test.

Similar to former studies, we replaced the linear term in the model with a B-spline function and established exposure-response (E-R) models for each city by setting two nodes at the 25th and 75th percentiles of the average PM concentration in all cities. Then, we used meta-analysis to combine and obtain the E-R curve for Jiangsu Province (15, 20).

We conducted sensitivity analyses spanning the period 2015–2019. The aim was to mitigate the potential confounding impact of the COVID-19 pandemic and China’s quarantine policy on both the levels of PM pollution and the associations observed between such pollution and CVD mortality.

All analyses were conducted with R 4.2.1 (R Foundation for Statistical Computing), using the mgcv and tlnise packages. Results are presented as the pooled percent change in mortality associated with a 10-μg/m^3^ increase in PM concentration. Statistical significance was considered at *p* < 0.05.

## 3 Results

**Figure 2:**
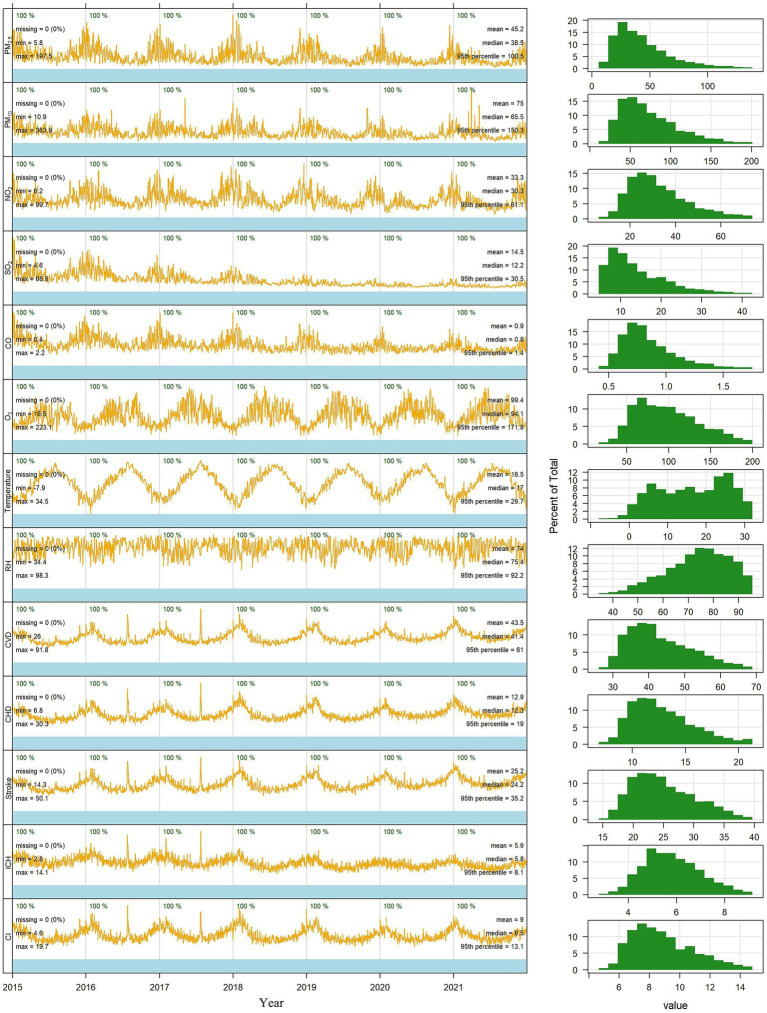
Time trends in air pollution data, meteorological data, and average daily cardiovascular disease mortality in 13 cities in Jiangsu, 2015–2021 (PM_2.5_, particulate matter <2.5 μm diameter; PM_10_, particulate matter <10 μm diameter; NO_2_, nitrogen dioxide; SO_2_, sulfur dioxide; CO, carbon monoxide; O_3_, ozone; RH, relative humidity; CVD, all cardiovascular disease; CHD, coronary heart disease; ICH, intracerebral hemorrhage; CI, cerebral infarction; Min, minimal value; Max, maximum value).

Figure 2 summarizes the descriptive statistics of daily environmental data and daily cause-specific cardiovascular disease mortality in Jiangsu Province between 2015 and 2021. The average daily mean concentrations in the 13 cities were 45.15 μg/m^3^ (median: 38.53 μg/m^3^ [range: 5.82, 197.51]) for PM_2.5_ and 75.03 μg/m^3^ (median: 65.47 μg/m^3^ [range: 10.93, 363.94]) for PM_10_. For meteorological variables, the daily mean temperature was 16.53°C (median: 17.00°C [range: −7.93, 34.48]), and the mean RH was 74.02% (median: 75.38% [range: 34.38, 98.27]). We recorded a daily average of 41.38 deaths per city for all cardiovascular diseases. Summary statistics for subcity and district environmental data are shown in Supplementary Table S1.

The PM_10_ concentration was strongly correlated with the PM_2.5_ concentration, with a mean Spearman correlation coefficient of 0.90. PM_2.5_ and PM_10_ concentrations were positively correlated with NO_2_, SO_2_, and CO concentrations, with correlation coefficients ranging from 0.570 to 0.714 (*p* ≤ 0.01). Mean temperature and RH were negatively correlated with primary pollutants (PM_2.5_, PM_10_, NO_2_, SO_2_, CO). Correlations between other pollutants are summarized in Supplementary Table S2.

We observed positive and significant associations between PM_2.5_ and PM_10_ concentrations and cause-specific CVD mortality in Jiangsu. The estimated association between PM_2.5_ and mortality (Table 1) was highest at lag 04 day (4-day moving average) for CVD, lag 03 day for CHD, stroke, and CI, and lag 02 day for ICH. Each 10-μg/m^3^ increase in PM_2.5_ concentration was associated with a 0.723% [95% confidence interval (CI): 0.512, 0.935] increase in daily CVD mortality, a 0.669% (95% CI: 0.461, 0.878) increase in CHD mortality, a 0.758% (95% CI: 0.584, 0.931) increase in stroke mortality, a 0.512% (95% CI: 0.245, 0.780) increase in ICH mortality, and a 0.876% (95% CI: 0.637, 1.116) increase in CI mortality.

**Table 1:** Pooled percent change (%) and 95% confidence intervals (CIs) in cause-specific CVD mortality per 10-μg/m^3^ increase in PM_2.5_ concentration at different lag days in Jiangsu Province.

Lag days	CVD	CHD	Stroke	ICH	CI
lag0	0.421 (0.334, 0.508)	0.476 (0.334, 0.618)	0.413 (0.298, 0.528)	0.346 (0.142, 0.550)	0.442 (0.258, 0.625)
lag1	0.405 (0.302, 0.507)	0.388 (0.246, 0.530)	0.431 (0.301, 0.562)	0.412 (0.208, 0.616)	0.441 (0.263, 0.619)
lag2	0.298 (0.188, 0.408)	0.235 (0.093, 0.377)	0.345 (0.209, 0.481)	0.231 (0.013, 0.451)	0.430 (0.265, 0.595)
lag3	0.238 (0.090, 0.386)	0.179 (0.022, 0.337)	0.264 (0.115, 0.413)	0.113 (−0.092, 0.317)	0.281 (0.116, 0.447)
lag4	0.112 (−0.012, 0.236)	0.089 (−0.077, 0.256)	0.080 (−0.072, 0.231)	−0.005 (−0.236, 0.226)	0.040 (−0.126, 0.207)
lag01	0.554 (0.447, 0.661)	0.578 (0.415, 0.742)	0.566 (0.433, 0.699)	0.494 (0.259, 0.730)	0.591 (0.373, 0.809)
lag02	0.648 (0.521, 0.775)	0.638 (0.453, 0.822)	0.685 (0.525, 0.845)	0.512 (0.245, 0.780)	0.771 (0.522, 1.021)
lag03	0.712 (0.541, 0.884)	0.669 (0.461, 0.878)	0.758 (0.584, 0.931)	0.484 (0.186, 0.782)	0.876 (0.637, 1.116)
lag04	0.723 (0.512, 0.935)	0.663 (0.426, 0.900)	0.753 (0.536, 0.970)	0.430 (0.071, 0.790)	0.841 (0.578, 1.105)

CVD, all cardiovascular disease; CHD, coronary heart disease; ICH, intracerebral hemorrhage; CI, cerebral infarction.

The effect of PM_10_ was most substantial for CVD, CHD, stroke, and ICH mortality at lag 04 day, whereas at lag 03 day, there was a larger percent change in CI mortality (Table 2). An increase of 10 μg/m^3^ in the PM_10_ concentration was associated with a 0.424% (95% CI: 0.293, 0.556) increase in the pooled estimate of CVD mortality, a 0.415% (95% CI: 0.228, 0.602) increase in CHD mortality, a 0.444% (95% CI: 0.330, 0.559) increase in stroke mortality, a 0.276% (95% CI: 0.026, 0.526) increase in ICH mortality and a 0.510% (95% CI: 0.353, 0.667) increase in CI mortality. These moving averages were then applied in subsequent analyses.

**Table 2:** Pooled percent change (%) and 95% confidence intervals (CIs) in cause-specific CVD mortality per 10-μg/m^3^ increase in PM_10_ concentration at different lag days in Jiangsu Province.

Lag days	CVD	CHD	Stroke	ICH	CI
lag0	0.204 (0.146, 0.261)	0.262 (0.145, 0.378)	0.208 (0.136, 0.281)	0.123 (−0.012, 0.258)	0.242 (0.127, 0.356)
lag1	0.197 (0.130, 0.264)	0.175 (0.075, 0.275)	0.223 (0.151, 0.294)	0.169 (0.034, 0.305)	0.246 (0.117, 0.375)
lag2	0.184 (0.111, 0.256)	0.162 (0.069, 0.256)	0.205 (0.122, 0.288)	0.151 (−0.015, 0.317)	0.240 (0.132, 0.348)
lag3	0.160 (0.068, 0.253)	0.143 (0.027, 0.258)	0.179 (0.088, 0.270)	0.097 (−0.039, 0.233)	0.186 (0.077, 0.294)
lag4	0.070 (0.001, 0.140)	0.048 (−0.060, 0.155)	0.053 (−0.032, 0.137)	0.073 (−0.111, 0.257)	0.027 (−0.083, 0.137)
lag01	0.264 (0.196, 0.333)	0.297 (0.169, 0.426)	0.284 (0.203, 0.366)	0.191 (0.034, 0.347)	0.326 (0.186, 0.465)
lag02	0.337 (0.257, 0.418)	0.339 (0.211, 0.466)	0.365 (0.273, 0.458)	0.234 (0.057, 0.412)	0.430 (0.276, 0.585)
lag03	0.402 (0.293, 0.511)	0.404 (0.243, 0.567)	0.435 (0.332, 0.538)	0.257 (0.051, 0.463)	0.510 (0.353, 0.667)
lag04	0.424 (0.293, 0.556)	0.415 (0.228, 0.602)	0.444 (0.330, 0.559)	0.276 (0.026, 0.526)	0.504 (0.331, 0.677)

CVD, all cardiovascular disease; CHD, coronary heart disease; ICH, intracerebral hemorrhage; CI, cerebral infarction.

Table 3 shows the estimates of percent changes in cause-specific CVD mortality attributable to each 10-μg/m^3^ change in PM concentration in the two-pollutant models. After adjustment for gaseous pollutants (NO_2_, SO_2_, CO), the magnitude of the association between PM concentrations fluctuated, but the differences were not statistically significant. However, the association between PM_10_ and CI mortality was significantly decreased after adjusting for NO_2_. Additionally, the association between PM and daily ICH mortality attenuated to be nonsignificant after adjusting for gaseous pollutants.

**Table 3:** Pooled percent change (%) and 95% confidence intervals (CIs) in cause-specific CVD mortality per 10-μg/m^3^ increase in PM concentration in two-pollutant models.

Two-pollutant	CVD	CHD	Stroke	ICH	CI
PM_2.5_	0.723 (0.512, 0.935)	0.669 (0.461, 0.878)	0.758 (0.584, 0.931)	0.512 (0.245, 0.780)	0.876 (0.637, 1.116)
PM_2.5_ + NO_2_	0.619 (0.302, 0.936)	0.748 (0.411, 1.087)	0.419 (0.061, 0.780)	0.364 (−0.007, 0.737)	0.421 (0.018, 0.826)
PM_2.5_ + SO_2_	0.584 (0.386, 0.783)	0.552 (0.302, 0.802)	0.619 (0.434, 0.805)	0.313 (−0.005, 0.633)	0.779 (0.495, 1.064)
PM_2.5_ + CO	0.580 (0.304, 0.857)	0.493 (0.156, 0.831)	0.711 (0.420, 1.004)	0.287 (−0.214, 0.791)	0.839 (0.415, 1.265)
PM_10_	0.424 (0.293, 0.556)	0.415 (0.228, 0.602)	0.444 (0.330, 0.559)	0.276 (0.026, 0.526)	0.510 (0.353, 0.667)
PM_10_ + NO_2_	0.286 (0.127, 0.445)	0.466 (0.220, 0.712)	0.244 (0.075, 0.414)	0.317 (0.036, 0.599)	0.250 (0.050, 0.449) ^*^
PM_10_ + SO_2_	0.309 (0.180, 0.438)	0.286 (0.114, 0.457)	0.346 (0.216, 0.475)	0.191 (−0.076, 0.460)	0.422 (0.245, 0.600)
PM_10_ + CO	0.244 (0.117, 0.371)	0.201 (0.002, 0.401)	0.331 (0.174, 0.488)	0.135 (−0.206, 0.477)	0.390 (0.160, 0.620)

CVD, all cardiovascular disease; CHD, coronary heart disease; ICH, intracerebral hemorrhage; CI, cerebral infarction. ^*^Statistical significant difference with the single pollutant model compared by a paired z-test (*p* < 0.05).

**Figure 3:**
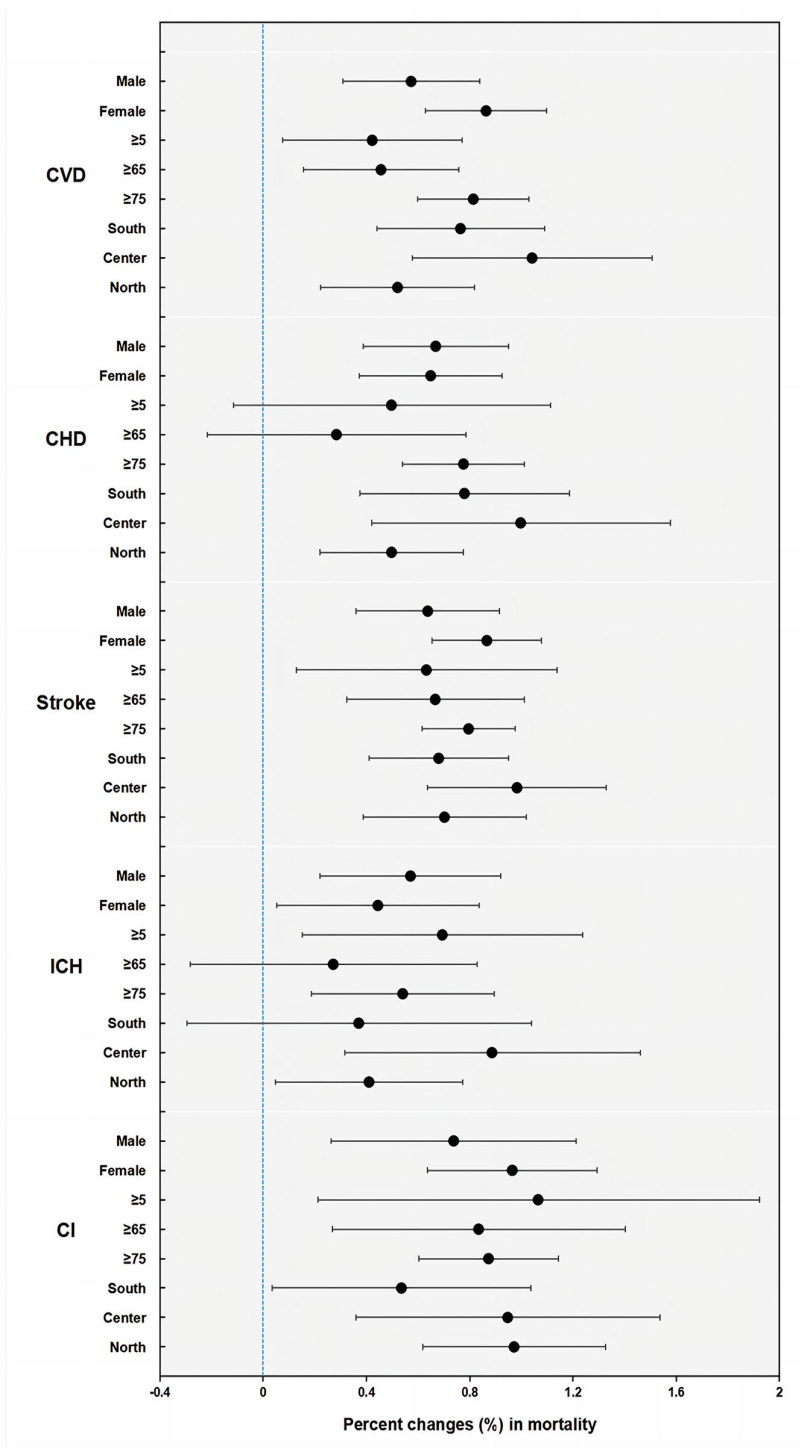
Percent changes (%) and 95% confidence intervals (CIs) (on the x-axis) in cause-specific CVD mortality by sex, age, and district per 10-μg/m^3^ increment of PM_2.5_ concentrations in Jiangsu (CVD, all cardiovascular disease; CHD, coronary heart disease; ICH, intracerebral hemorrhage; CI, cerebral infarction).

**Figure 4:**
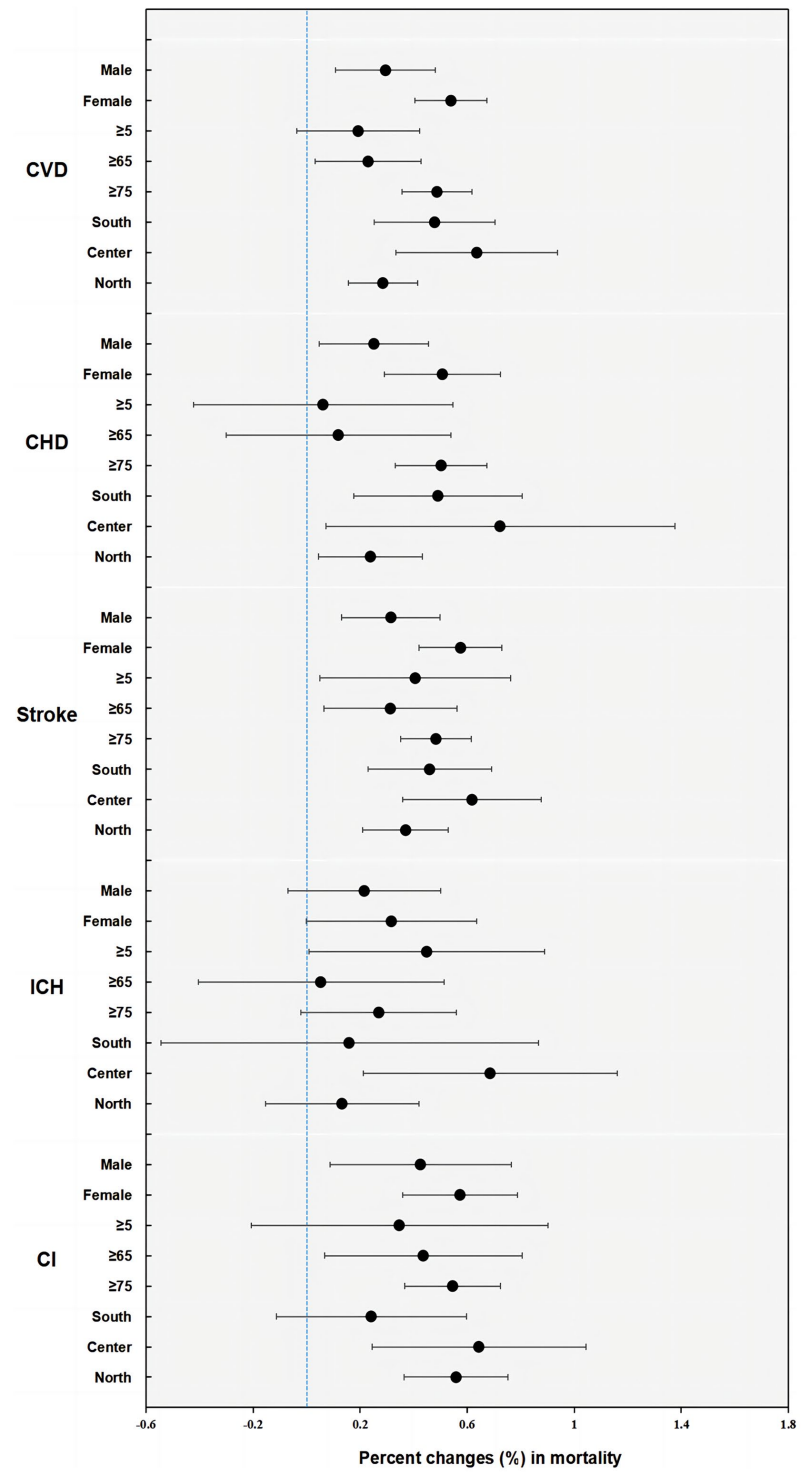
Percent changes (%) and 95% confidence intervals (CIs) (on the x-axis) in cause-specific CVD mortality by sex, age, and district per 10-μg/m^3^ increment of PM_10_ concentrations in Jiangsu (CVD, all cardiovascular disease; CHD, coronary heart disease; ICH, intracerebral hemorrhage; CI, cerebral infarction).

The results of stratified analyses by sex, age, and district based on pooled estimates of 13 cities in Jiangsu are presented in Figures 3, 4, and Supplementary Table S3. We found a stronger effect of PM_10_ in females (*p* = 0.038), with an increase of 0.295% (95% CI: 0.108, 0.481) for males and 0.539% (95% CI: 0.404, 0.674) for females. For age groups, the excess risk for CVD mortality per 10-μg/m^3^ increase in PM_10_ concentration was higher among older adults aged over 75 (*p* = 0.029), at 0.487% (95% CI: 0.356, 0.617). For districts, the effect size in Central Jiangsu was much larger than that in Northern Jiangsu (*p* = 0.036), with 0.635% (95% CI: 0.334, 0.937) and 0.284% (95% CI: 0.155, 0.414), respectively (shown in Figure 4). Similar differences between sex, age, and districts were shown in the PM_2.5_-and-CVD-mortality association, but with no statistical significance (shown in Figure 3).

**Figure 5:**
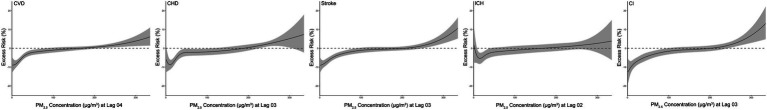
Pooled exposure-response relationship curves for the association of PM_2.5_ with daily cause-specific CVD mortality. The x-axis is the concentration of PM_2.5_ on lag days; the y-axis can be interpreted as the relative change in mortality. The solid line represents the mean estimate, the shaded area represents the 95% CI, and the dashed line represents an excess risk of 0%.

**Figure 6:**
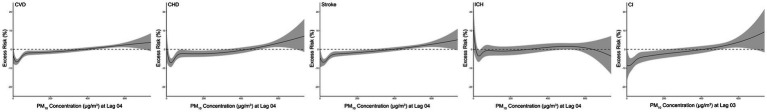
Pooled exposure-response relationship curves for the association of PM_10_ with daily cause-specific CVD mortality. The x-axis is the concentration of PM_10_ on lag days; the y-axis can be interpreted as the relative change in mortality. The solid line represents the mean estimate, the shaded area represents the 95% CI, and the dashed line represents an excess risk of 0%.

Figures 5, 6 show the E–R relationship curves for PM_2.5_ and PM_10_ concentrations and cause-specific CVD mortality. The E-R curve for PM_2.5_ and CVD mortality (shown in Figure 5) indicates increased risks, with a sharp slope at approximately 40 μg/m^3^, a moderate slope at 40–200 μg/m^3^, and a leveling with much wider confidence intervals at greater than 200 μg/m^3^. For PM_10_ (shown in Figure 6), the curve is slightly nonlinear, with a slight fluctuation followed by a sharp slope at concentrations below 85 μg/m^3^, then attenuation at exposure ranges lower than 550 μg/m^3^, and eventually leveling off with wider confidence intervals afterward.

Considering the potential influence of the COVID-19 pandemic and relative policies in 2020 on both the level of PM pollution and the incidence of CVD mortality, we conducted sensitivity analyses by using the data between 2015 and 2019 (shown in Supplementary Table S4). Similar to the main results, the estimated association between PM and mortality was highest at lag 04 day for CVD, and ERs between 2015 and 2019 were slightly lower than the overall estimates.

## 4 Discussion

In this study, we thoroughly analyzed multisite data on air pollution and cause-specific CVD mortality in Jiangsu Province from 2015 to 2021. By using a common protocol to analyze the data and by conducting the study over a long period, our pooled estimates of the percentage change in mortality are more representative and less susceptible to publication bias. Our findings provide further evidence to support the association between short-term exposure to PM and increased cardiovascular disease mortality in developing countries, with a higher pollution level than in North America and Europe. The estimates remained generally consistent in sensitivity analyses when excluding the data during the COVID-19 pandemic period.

In the analysis of PM_2.5_, we observed that each 10-μg/m^3^ increase in PM_2.5_ concentration was associated with a 0.723% increase in all-cause cardiovascular disease mortality. Previous studies have shown a high degree of variability. Our estimate was slightly lower compared with multicity time-series studies from developed countries (14, 24–26). There were combined estimates of 1.03% in 75 U.S. cities per 10-μg/m^3^ (14), 0.84% in North America and Europe (25), and 0.86% in 12 Mediterranean cities (26). However, our estimate is much higher than that of a study conducted in 652 cities mainly located in East Asia (20), with an increase of 0.55% from cardiovascular diseases. Additionally, increments of 0.44% (27) and 0.27% (15) in all-cause CVD mortality were reported in research conducted in China.

Our analysis of PM_10_ revealed a 0.424% increase in all-cause CVD mortality for every 10-μg/m^3^ increase in PM_10_ concentration, which is generally consistent with an umbrella review interval of 0.39% to 0.49% (28). Other studies reported excess risks of 0.9% and 0.12% in daily all-cause CVD mortality in France and Spain (29) and 0.44% from 4 Asian cities of the PAPA project (30). The combined excess risks of CVD mortality were 0.36% (31) and 0.49% (32) for each 10-μg/m^3^ increase in PM_10_ concentration among the Chinese population.

There is a large variation in estimates between studies and heterogeneity across regions of China. One possible explanation for the variation in estimates is the shape of the E-R curve. Similar to previous studies, our study also suggested that the curve flattens out at higher concentrations, indicating a harvesting effect (33). This means that long-term exposure to high levels of ambient PM could lead to potential mortality displacement for Chinese residents, and the impact of PM may be underestimated (34, 35). Second, differences in climate and outdoor activity patterns across regions may contribute to the variation (26, 36, 37). For instance, cold weather can restrict people from being outdoors, where pollution monitoring sites are set, leading to an increased error in the effect (37). Conversely, Mediterranean-type climate regions shared similar estimates in all-cause mortality (24). Third, although our data on the composition of PM and its relative toxicity are insufficient, previous studies suggest that PM in China is mainly derived from crustal constituents or dust (38), which may be slightly less toxic than those derived mainly from fossil combustion (39).

Our study, along with earlier works, supports the evidence that PM_2.5_ is more hazardous than PM_10_ (20, 31). First, the smaller size of PM_2.5_ particles enables deeper penetration into the respiratory system and bloodstream (40, 41). The uptake of PM by alveolar macrophages then plays a crucial role in triggering pulmonary inflammation and oxidative stress following exposure to PM (42, 43). These processes can damage the inner lining of blood vessels, leading to the formation of atherosclerotic plaques and an increased risk of CVD (44, 45). Additionally, PM_2.5_ contains a higher concentration of toxic pollutants, such as heavy metals and organic compounds, which further harm the cardiovascular system (46). Furthermore, compared to PM_10_, PM_2.5_ exposure has a stronger link to increased systolic blood pressure and a higher risk of hypertension, which can also contribute to CVD (47).

Although the effect size on cardiovascular disease mortality slightly decreased after adding gaseous pollutants to the model, the association remained statistically significant, suggesting an independent health effect of PM. Notably, the estimates in the two-pollutant models differed across cardiovascular disease subtypes. Previous studies on PM and stroke have also shown that fine particles absorbed in the respiratory tract can be transferred to the central nervous system via the blood–brain barrier (BBB). Meanwhile, pollutants can disrupt the BBB by altering cerebral microvascular integrity. Physical properties or associated toxic compounds may contribute to the development of stroke (48, 49). Our study also found a greater effect of PM on ischemic strokes than hemorrhagic strokes, as was observed in some cohort studies (50, 51). The mechanism may be explained by the following hypotheses. Free radicals from air pollutants may cause an inflammatory response that enhances blood coagulation and plasma viscosity. This would therefore increase susceptibility to ischemic strokes rather than hemorrhagic strokes (52). In addition, the interpretations of the results for intracerebral hemorrhage mortality are impaired by the low number of cases in our cities, as the estimates provide a wider confidence interval.

Consistent with other studies, we reported that women and older adults were more likely to be susceptible to ambient PM (20). Exposure, absorption, and degradation of pollutants vary by sex (53, 54). Some studies have also suggested that hormonal changes during the menstrual cycle may play a role in women’s increased vulnerability to PM. For older people, the sensitivity may be due to their reduced ability to clear pollutants from their cardiorespiratory system and a higher prevalence of underlying cardiovascular diseases. In district-specific analysis, we found weaker associations with daily CVD mortality in the district with higher PM concentrations, as reported in prior research (15, 20, 55). This can be interpreted by the E-R curve and the harvesting effect. Additionally, enhanced public health policies and personal interventions in highly polluted areas may lead to reduced exposure. On the other hand, when we compared the estimates in two regions that had significant economic disparities – Southern and Northern Jiangsu, we found no significant difference. A systematic review reveals that, despite limited research exploring the effect modification of the relationship between air pollution and CVD outcomes by socioeconomic position, it is evident that there are identifiable gaps and inconsistencies (56).

This study has certain limitations. First, pollutant concentration data from fixed environmental monitoring stations were used to represent the average exposure level of the population. It is possible, however, that the true exposure level of the population may be misclassified since people spend the majority of their time indoors and breathe mainly in the breathing zone. Second, although our strict quality control procedures have minimized the possibility, diagnostic errors or coding errors are inevitable in long-term studies based on mortality registration data. Third, as in other ecologic studies, ecological fallacy cannot be avoided. Further cohort studies are needed to investigate the underlying mechanisms and individual-level effects of ambient particulate air pollution.

## 5 Conclusion

In conclusion, our time-series study provides evidence of positive associations between acute exposure to PM_2.5_ and PM_10_ and total and cause-specific cardiovascular disease mortality in Jiangsu, eastern China. The associations remained robust after adjusting for gaseous pollutants. Females, older adults and districts with lower average PM levels are more sensitive, especially for PM_10_. The E-R curve for PM on CVD mortality is steeper at lower concentrations and flattens out at higher concentrations. Our study is an update and addition to the evidence on the acute circulatory system effects of PM in developing countries and provides a basis for further research and environmental policy formation.

## Data availability statement

The raw data supporting the conclusions of this article will be made available by the authors, without undue reservation.

## Author contributions

FZ, HY, XF, ZD, QW, and JZ contributed to the study conception and design. Material preparation and data collection were performed by HY, ZD, and QW. Data curation and analysis were performed by FZ and the first draft of the manuscript was written by FZ. XF and JZ revised the manuscript. All authors contributed to the article and approved the submitted version.
