# Supplementary material for: Particulate air pollution and cardiovascular disease mortality in Jiangsu Province, China: a time-series analysis between 2015 and 2021
Source: Front Public Health. 2023 Dec 20;11:1218479. doi: 10.3389/fpubh.2023.1218479 (PMC10761421; doi:10.3389/fpubh.2023.1218479)
Supplement: Supplementary file 1 [file Data_Sheet_1.pdf]

Supplementary Material

Particulate air pollution and cardiovascular disease mortality in Jiangsu Province, China: A time-series analysis between 2015 and 2021

Fangyu Zhu, Hao Yu, Xikang Fan, Zhen Ding, Qingqing Wang, Jinyi Zhou*

***Correspondence:** Jinyi Zhou: zhoujinyi74@sina.com

1 Supplementary Tables

Table S1. Descriptive statistics for subcity and district environmental data in Jiangsu, from 2015 to 2021[a]

	$PM_{2.5}$, μg/m^3	PM_{10}, μg/m^3	NO_2, μg/m^3	SO_2, μg/m^3	CO, mg/m^3	O_3, μg/m^3	Temperature, ℃	RH, %
**Southern Jiangsu**	36.40(24.36,55.50)	63.33(43.90,93.00)	35.44(26.50,47.93)	10.77(7.50,16.86)	0.81(0.66,0.99)	92.67(65.00,133.14)	17.90(9.40,25.00)	74.00(64.00,84.00)
Nanjing	34.29(22.93,51.86)	64.93(45.08,94.43)	34.07(25.93,46.57)	10.36(7.36,15.79)	0.83(0.70,1.01)	94.00(65.00,132.64)	17.50(9.10,24.60)	75.00(64.00,85.00)
Wuxi	36.50(24.50,54.43)	63.00(44.94,90.86)	37.25(28.19,49.75)	10.61(7.77,16.14)	0.86(0.71,1.09)	89.57(61.75,132.31)	18.00(9.70,25.10)	74.00(64.00,83.00)
Changzhou	38.90(26.21,57.30)	63.60(44.20,93.30)	34.90(26.30,47.00)	12.30(9.30,18.30)	0.86(0.72,1.04)	95.20(65.64,137.50)	17.90(9.30,25.10)	74.00(64.00,84.00)
Suzhou	32.50(22.07,51.89)	59.00(40.44,87.78)	37.70(28.44,50.40)	9.20(6.69,15.30)	0.71(0.57,0.89)	90.60(64.70,130.88)	18.40(10.10,25.10)	74.00(65.00,83.00)
Zhenjiang	39.75(26.25,61.10)	66.75(45.50,98.75)	32.80(24.00,44.80)	10.75(7.20,18.50)	0.76(0.62,0.92)	93.00(68.00,132.60)	17.60(9.00,24.80)	75.00(65.00,85.00)
**Central Jiangsu**	34.50(22.67,53.28)	59.17(40.25,89.50)	26.50(18.71,38.37)	12.14(8.40,18.00)	0.79(0.63,0.99)	95.33(71.38,128.26)	17.00(8.60,24.60)	79.00(69.00,88.00)
Nantong [b]	31.33(20.25,48.80)	49.83(35.00,76.20)	26.60(17.00,42.00)	12.67(8.20,18.00)	0.63(0.50,0.80)	98.71(77.08,130.24)	17.20(9.10,24.60)	80.00(70.00,89.00)

Yangzhou	37.95(24.75,56.50)	67.75(46.38,99.50)	27.13(20.00,37.57)	13.00(8.75,20.46)	0.81(0.67,1.01)	90.28(65.93,125.25)	17.00(8.45,24.60)	77.00(67.00,85.00)
Taizhou [b]	34.56(22.79,53.40)	59.77(40.91,91.29)	25.60(18.21,36.17)	11.22(8.13,15.50)	0.88(0.74,1.10)	96.50(71.71,129.41)	16.70(8.40,24.60)	80.00(70.79,89.00)
**Northern Jiangsu**	37.50(23.50,60.00)	65.93(43.40,100.00)	25.44(17.75,36.86)	11.60(7.97,17.91)	0.79(0.63,1.02)	89.20(62.80,122.67)	16.30(7.30,24.10)	74.50(62.00,84.00)
Xuzhou	49.28(33.43,74.89)	90.57(62.71,128.88)	35.71(25.29,48.73)	15.40(9.86,26.43)	0.89(0.69,1.20)	96.53(63.71,136.60)	16.60(7.00,24.80)	69.00(56.00,79.00)
Lianyungang	29.00(17.50,49.50)	52.50(33.67,82.33)	26.50(19.00,38.33)	11.50(7.50,17.86)	0.78(0.61,0.99)	89.33(56.75,123.25)	16.20(7.10,23.70)	73.00(60.00,83.00)
Huaian	38.89(24.50,63.67)	61.73(41.89,91.50)	22.20(16.22,31.00)	10.88(7.64,15.89)	0.84(0.69,1.08)	87.10(62.00,119.73)	16.10(7.30,23.80)	78.00(68.00,86.70)
Yancheng	30.92(19.67,51.07)	59.23(39.91,93.58)	20.25(14.57,29.83)	9.96(7.09,14.40)	0.66(0.54,0.83)	83.00(63.50,106.17)	16.40(7.70,23.90)	77.00(67.00,85.00)
Suqian	38.50(26.13,58.63)	67.83(47.86,99.29)	25.00(17.71,34.67)	11.86(8.00,17.63)	0.78(0.62,1.03)	94.40(66.75,129.14)	16.50(7.40,24.30)	74.00(62.00,83.75)

[a] Average levels were presented as median (P_{25}, P_{75}); $PM_{2.5}$, particulate matter <2.5 μm diameter; PM_{10}, particulate matter <10 μm diameter; NO_2, nitrogen dioxide; SO_2, sulfur dioxide; CO, carbon monoxide; O_3, ozone; RH, relative humidity

[b] Time period from 2016 through 2021

Table S2. The Spearman correlation between pollutants and meteorology variables [a]

	$PM_{2.5}$	PM_{10}	NO_2	SO_2	CO	O_3	Temperature, ℃	RH, %
$PM_{2.5}$	1	0.897 *	0.651 *	0.570 *	0.714 *	-0.045 *	-0.414 *	-0.187 *
PM_{10}		1	0.673 *	0.609 *	0.625 *	0.031 *	-0.354 *	-0.376 *
NO_2			1	0.507 *	0.569 *	-0.120 *	-0.368 *	-0.268 *
SO_2				1	0.550 *	0.027 *	-0.196 *	-0.328 *
CO					1	-0.125 *	-0.304 *	-0.038 *
O_3						1	0.571 *	-0.298 *
Temperature, ℃							1	0.151 *
RH, %								1

[a] $PM_{2.5}$, particulate matter <2.5 μm diameter; PM_{10}, particulate matter <10 μm diameter; NO_2, nitrogen dioxide; SO_2, sulfur dioxide; CO, carbon monoxide; O_3, ozone; RH, relative humidity

* $P<0.05$

Table S3. Pooled percent change (%) and 95% confidence intervals (CIs) in CVD mortality per 10-μg/m^3 increase in PM concentration stratified by sex, age, and district [a]

	$PM_{2.5}$		PM_{10}	
	Pooled estimate%(95%CI)	*P*-value for difference [b]	Pooled estimate%(95%CI)	*P*-value for difference [b]
**Sex**				
Male	0.573(0.307,0.839)	ref	0.295(0.108,0.481)	ref
Female	0.863(0.629,1.098)	0.108	0.539(0.404,0.674)	0.038 [*]
**Age**				
5-64 years	0.422(0.075,0.771)	ref	0.192(-0.036,0.422)	ref
65-74 years	0.456(0.156,0.758)	0.885	0.229(0.032,0.427)	0.809
>=75 years	0.814(0.599,1.029)	0.061	0.487(0.356,0.617)	0.029 [*]
**District**				
Southern Jiangsu	0.764(0.439,1.090)	0.279	0.478(0.253,0.703)	0.143
Central Jiangsu	1.041(0.578,1.507)	0.064	0.635(0.334,0.937)	0.036 [*]
Northern Jiangsu	0.520(0.223,0.819)	ref	0.284(0.155,0.414)	ref

[a] $PM_{2.5}$, particulate matter <2.5 μm diameter; PM_{10}, particulate matter <10 μm diameter
[b] The *P*-value for difference was compared by a paired z-test
[*] $P<0.05$

Table S4. Pooled percent change (%) and 95% confidence intervals (CIs) in CVD mortality per 10-μg/m3 increase in PM2.5 and PM10 concentration at different lag days in Jiangsu Province from 2015 to 2019. [a]

Lag days	$PM_{2.5}$	PM_{10}
lag0	0.365(0.254,0.476)	0.196(0.124,0.269)
lag1	0.402(0.261,0.544)	0.209(0.113,0.304)
lag2	0.295(0.175,0.415)	0.163(0.087,0.239)
lag3	0.246(0.078,0.414)	0.156(0.046,0.266)
lag4	0.119(-0.010,0.249)	0.080(-0.004,0.164)
lag01	0.496(0.342,0.650)	0.260(0.160,0.359)
lag02	0.576(0.412,0.741)	0.311(0.207,0.416)
lag03	0.649(0.454,0.844)	0.372(0.248,0.497)
lag04	0.666(0.455,0.876)	0.397(0.259,0.536)

[a] CVD, all cardiovascular disease
